# Marine Sponge and Octocoral-Associated Bacteria Show Versatile Secondary Metabolite Biosynthesis Potential and Antimicrobial Activities against Human Pathogens

**DOI:** 10.3390/md21010034

**Published:** 2022-12-30

**Authors:** João F. Almeida, Matilde Marques, Vanessa Oliveira, Conceição Egas, Dalila Mil-Homens, Romeu Viana, Daniel F. R. Cleary, Yusheng M. Huang, Arsénio M. Fialho, Miguel C. Teixeira, Newton C. M. Gomes, Rodrigo Costa, Tina Keller-Costa

**Affiliations:** 1 iBB—Institute for Bioengineering and Biosciences and i4HB—Institute for Health and Bioeconomy, Instituto Superior Técnico, Av. Rovisco Pais, 1049-001 Lisbon, Portugal; 2 Department of Bioengineering, Instituto Superior Técnico, Av. Rovisco Pais, 1049-001 Lisbon, Portugal; 3 Department of Biology and Centre for Environmental and Marine Studies (CESAM), University of Aveiro, 3810-193 Aveiro, Portugal; 4 Center for Neuroscience and Cell Biology (CNC), Rua Larga—Faculdade de Medicina, University of Coimbra, 3004-504 Coimbra, Portugal; 5 Biocant—Transfer Technology Association, BiocantPark, 3060-197 Cantanhede, Portugal; 6 Department of Marine Recreation, National Penghu University of Science and Technology, Magong City 880-011, Taiwan; 7 Centre of Marine Sciences (CCMAR/CIMAR LA), University of Algarve, Campus de Gambelas, 8005-139 Faro, Portugal

**Keywords:** bioprospection, biosynthetic gene clusters, blue biotechnology, culture collections, genomics, marine bacteria

## Abstract

Marine microbiomes are prolific sources of bioactive natural products of potential pharmaceutical value. This study inspected two culture collections comprising 919 host-associated marine bacteria belonging to 55 genera and several thus-far unclassified lineages to identify isolates with potentially rich secondary metabolism and antimicrobial activities. Seventy representative isolates had their genomes mined for secondary metabolite biosynthetic gene clusters (SM-BGCs) and were screened for antimicrobial activities against four pathogenic bacteria and five pathogenic *Candida* strains. In total, 466 SM-BGCs were identified, with antimicrobial peptide- and polyketide synthase-related SM-BGCs being frequently detected. Only 38 SM-BGCs had similarities greater than 70% to SM-BGCs encoding known compounds, highlighting the potential biosynthetic novelty encoded by these genomes. Cross-streak assays showed that 33 of the 70 genome-sequenced isolates were active against at least one *Candida* species, while 44 isolates showed activity against at least one bacterial pathogen. Taxon-specific differences in antimicrobial activity among isolates suggested distinct molecules involved in antagonism against bacterial versus *Candida* pathogens. The here reported culture collections and genome-sequenced isolates constitute a valuable resource of understudied marine bacteria displaying antimicrobial activities and potential for the biosynthesis of novel secondary metabolites, holding promise for a future sustainable production of marine drug leads.

## 1. Introduction

Antibiotic resistant microorganisms emerged as a problem soon after the large-scale introduction of the first antibiotics [1]. At present, multi-drug resistant microbes pose a worldwide threat, with estimates placing their toll on human life as high as 700,000 deaths per year, with simulations predicting that this number will rise to 10 million per year by 2050 [1]. *Staphylococcus aureus*, part of the human skin microbiome and an opportunistic pathogen, is, for example, commonly resistant to penicillin, while methicillin-resistant *Staphylococcus aureus* (MRSA) strains show additional resistance to many antimicrobial drug classes, including oxacillin, cefoxitin and chloramphenicol [2]. Nosocomial infections by MRSA are frequent, with >150,000 cases per year registered in the European Union alone [3]. *Salmonella enterica* serovar Typhimurium [4] and *Vibrio parahaemolyticus* [5] are not only human pathogens, but also swine and seafood pathogens, respectively. *Salmonella* spp. are estimated to cause about 93 million cases of gastroenteritis and 155,000 deaths annually, with serovar Typhimurium being one of the leading causes [6]. *S. enterica* serovar Typhimurium isolates from Southeast Asia were found to be commonly resistant to seven classes of antibiotics, including streptomycin, ampicillin and erythromycin [4]. *Vibrio parahaemolyticus* can have negative impacts on aquaculture production (at times leading to 100% loss in shrimp production [7]) and causes disease in fish, shellfish, and shrimp [7,8]. Likewise, the incidence of treatment–resistant fungi of the *Candida* genus has increased over the past 20 years [9]. Although *C. albicans* continues to be the most prevalent cause of human candidiasis, *C. glabrata*, *C. tropicalis* and *C. parapsilosis* have been gaining ground mainly due to their greater ability to exhibit antifungal drug resistance [9,10]. *Candida auris* is the most recent addition to this group, due to the extreme resistance that some strains have shown towards marketed drugs [11]. Indeed, certain *C. auris* strains are resistant to all approved antifungal drug classes and are now considered nearly untreatable [11]. Therefore, new drugs and a greater structural variety of natural products are urgently required to combat the antimicrobial resistance crisis [12]. 

Marine animals, such as sponges (Porifera) and octocorals (Cnidaria, Anthozoa, Octocorallia), are well-known producers of diverse natural products with antibacterial, antitumoral, antiviral, anti-inflammatory and other activities [13,14]. Several marine animal-derived compounds such as, e.g., the anti-cancer drugs eribulin (trade name Halaven) [15] and trabectedin (trade name Yondelis) [16], are already available on the market. Sponges and corals host diverse and distinct microbial communities, contributing to carbon, sulfur and nitrogen cycling as well chemical defense of their host [17,18]. Advances in next-generation sequencing technologies, metagenomics, and the development of bioinformatic tools, such as the “antibiotics and Secondary Metabolite Analysis Shell” (antiSMASH) [19], to mine genomes and metagenomes for secondary metabolite biosynthetic gene clusters (SM-BGCs), have contributed to the understanding that microbial symbionts are frequently the true producers of the bioactive compounds previously isolated from marine animals. For example, octocoral-derived bacteria and fungi have been shown to harbor an enormous arsenal of bioactive terpenes, steroids, alkaloids, and polyketides [13]. Recent metagenomic surveys found the marine sponge microbiome to be significantly enriched in polyketide synthase encoding genes [20], while a variety of non-ribosomal peptide synthetase gene clusters, among others, were reported from the microbiomes of octocorals [21]. Moreover, bacteria isolated from the marine sponges *Hymeniacidon perlevis* and *Halichondria panicea* were recently shown to inhibit MRSA growth [22]. Thus, the microbiomes of marine animals remain a largely untapped reservoir of novel molecules with unknown functions, some of which may be leveraged for pharmaceutical use. 

Microbial cultivation, though tedious and time-consuming and hence sometimes disregarded in the omics era, is a crucial component to harness the biotechnological and medical potential of microorganisms. The improvement of cultivation protocols, and development of innovative media compositions and culture conditions tailored for marine, host-associated bacteria are pivotal to successfully culture difficult-to-cultivate symbionts [23]. Well-tended, taxonomically diverse culture collections are a valuable resource for natural products research, and the isolation of new species and strains stands at the very basis of the marine drug discovery pipeline.

The goal of this study was to highlight novel bacterial isolates suitable for bioprospecting among two comprehensive culture collections totaling 919 marine, animal-associated bacteria. Moreover, we aimed to assess the abundance and diversity of biosynthetic gene clusters, particularly those encoding for novel polyketides and non-ribosomal and ribosomal peptides, in the genomes of selected isolates. Finally, we tested whether the bacterial isolates inhibit the growth of human pathogenic bacteria and fungi to select promising antimicrobial strains worth further investigation. The first collection, hereafter called the “MicroEcoEvo” collection, was established between 2010 and 2020 during multiple sampling events of temperate marine sponges [24,25,26], octocorals [27] and fish larvae [28,29,30] in Portugal, by employing various marine culture media and conditions. The second collection, hereafter called the “EcoTech-SPONGE” collection, comprises bacterial isolates from various tropical marine sponges collected off the coast of Taiwan in 2017 [31,32]. In-depth 16S rRNA gene phylogenetic assessments were carried out to characterize the diversity and taxonomic novelty of both collections. A panel of 70 isolates with sequenced genomes was then subjected to phylogenomic assessments and genome-wide screenings for SM-BGCs. Finally, we screened these isolates for antimicrobial activities against the bacterial pathogens *S. aureus*, MRSA, *S. enterica* serovar Typhimurium and *V. parahaemolyticus* and five fungal pathogens of the *Candida* genus, paving the way for future studies on the identification and structure elucidation of novel natural products of marine origin.

## 2. Results

### 2.1. Taxonomic and Phylogenetic Diversity of the Two Marine Culture Collections

**Figure 1:**
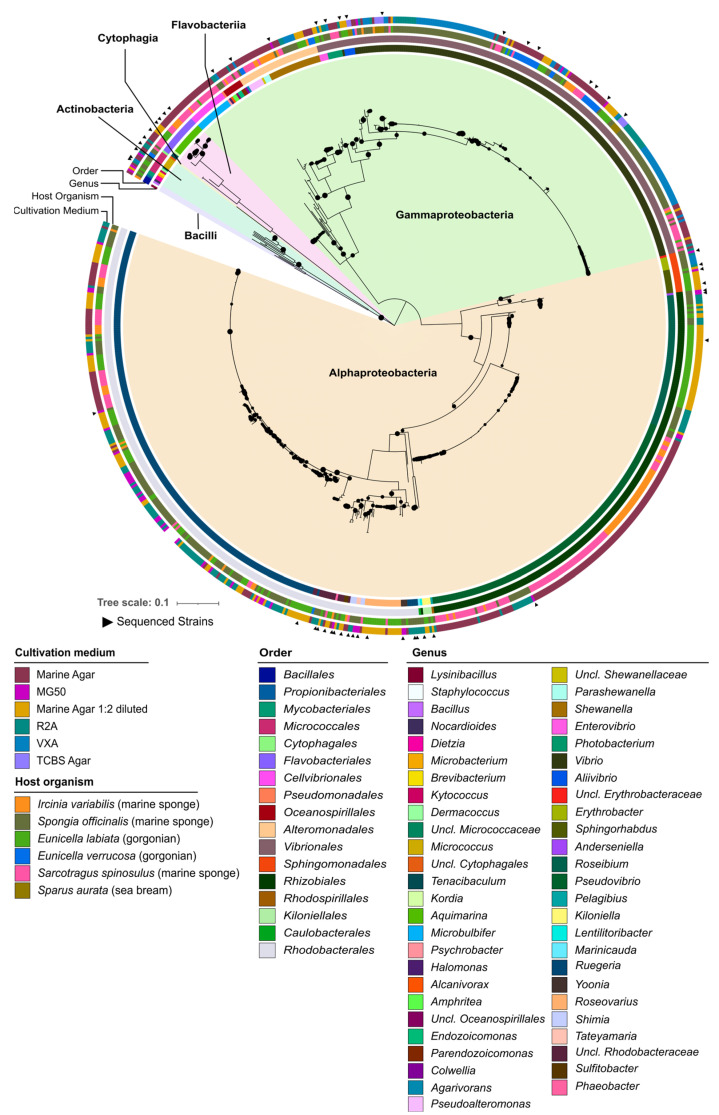
16S rRNA gene-based phylogenetic inference of the 800 coral-, sponge- and fish-associated bacteria from the “MicroEcoEvo” culture collection. Black triangles indicate isolates that had their genomes sequenced. The outer ring indicates, by color, the cultivation medium used for isolation and the intermediate outer ring, the host species of each isolate. The intermediate inner ring and the inner ring indicate order and genus-level taxonomic classifications of the isolates, respectively. The legends are organized from left to right by order of appearance in the tree. Black dots on the branches indicate bootstrap support of ≥70% (500 bootstrap repetitions). Uncl.—unclassified.

We determined the phylogenetic breadth of 919 bacterial strains, isolated from 18 marine sponge species (697 strains), two octocoral species (212 strains), and one fish species (10 strains) of two culture collections, the “MicroEcoEvo” collection (available at iBB, Instituto Superior Técnico, Lisbon Portugal) and the “EcoTech-SPONGE” collection (available at CESAM, Aveiro, Portugal). Complete lists of all isolates from both collections can be found in Tables S1 and S2 (Supplementary File S2), respectively. The “MicroEcoEvo” collection (Table S1) comprised 800 bacteria isolated from marine animals off the coast of Portugal spanning four bacterial phyla, six classes, and 45 genera (Figure 1). The most represented genera in this collection were *Ruegeria* (218 isolates), *Vibrio* (192 isolates), *Pseudovibrio* (140 isolates), *Roseibium* (62 isolates) and *Shewanella* (25 isolates). The collection also comprised less frequently cultured genera such as *Amphritea* (two isolates), *Endozoicomonas* (two isolates) and *Parendozoicomonas* (two isolates), as well as *Kordia*, *Anderseniella* and *Lentilitoribacter* (one isolate each) (Figure 1). 

Nineteen of the isolates in this collection were not classifiable at genus level according to the Ribosomal Database Project (RDP) taxonomy [33], and represent likely at least six novel genera in the families *Micrococcaceae*, *Rhodobacteraceae*, *Erythrobacteraceae* and *Shewanellaceae*, and the orders *Cytophagales* and *Oceanospirillales*, respectively (Figure 1).

**Figure 2:**
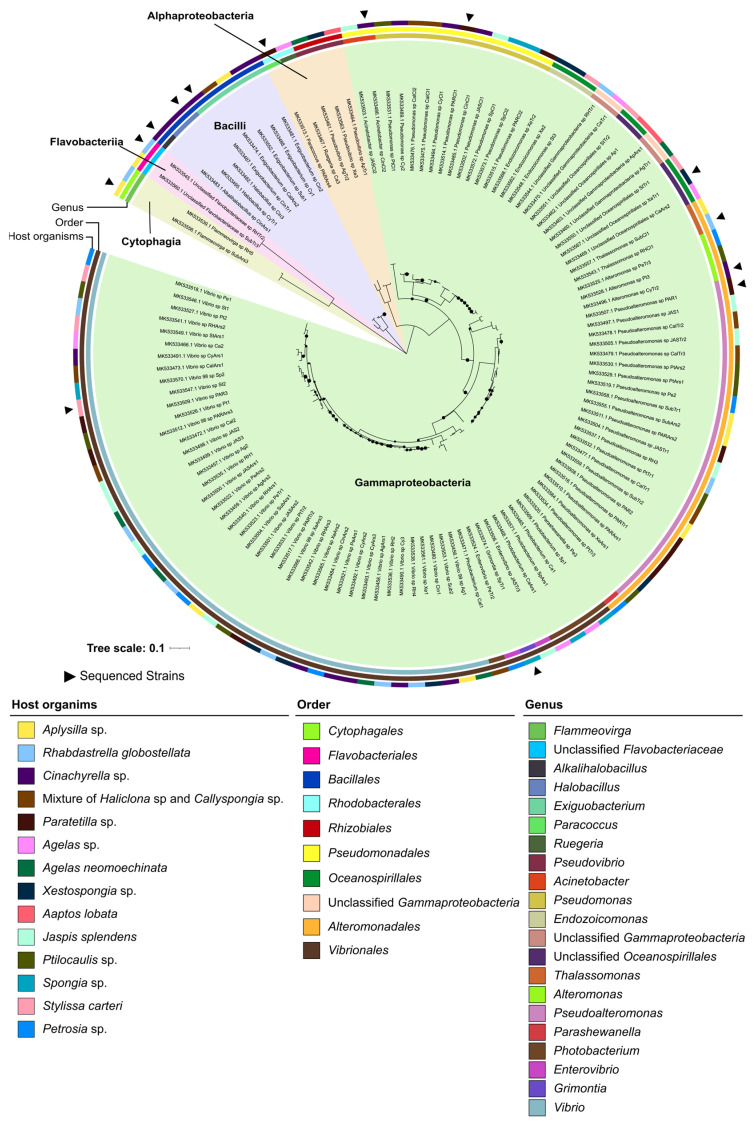
16S rRNA gene-based phylogenetic inference of the 119 sponge-associated bacteria from the “EcoTech-SPONGE” culture collection. Black triangles indicate isolates that had their genomes sequenced. The outer ring indicates, by color, the host species of the bacterial isolates. The intermediate and the inner ring show order and genus-level taxonomic classifications of the isolates, respectively. The legends are organized from left to right by order of appearance in the tree. Black dots on the branches indicate bootstrap support of ≥70% (500 bootstrap repetitions).

The “EcoTech-SPONGE” culture collection (Table S2) comprised 119 bacteria isolated from 15 marine sponge species and spanned three bacterial phyla, five classes and 18 genera (Figure 2). All isolates in this collection were cultivated on half strength (1:2 diluted) Marine Agar (MA) at 17 °C and the number of isolates per sponge species varied from three (from *Aaptos lobata*) to 17 (from *Cinachyrella sp*.). The isolates were mostly related to *Gammaproteobacteria* (102 of 119), with low numbers of *Bacilli* (eight isolates), *Alphaproteobacteria* (five isolates), *Cytophagia* and *Flavobacteriia* (two isolates each). The most represented genera were *Vibrio* (42 isolates), *Pseudoalteromonas* (21 isolates) and *Pseudomonas* (11 isolates) (Figure 2). Less frequently cultured genera were *Endozoicomonas* (3 isolates), *Thalassomonas* (2 isolates), *Flammeovirga* (2 isolates), and *Grimontia* (1 isolate) (Figure 2). In this collection, 11 isolates could not be classified at genus, family, or order level, possibly comprising novel taxa in the family *Flavobacteriaceae* (*n =* 2), the order *Oceanospirillales* (*n =* 5) and the class *Gammaproteobacteria* (*n =* 4), respectively (Figure 2). 

The “EcoTech-SPONGE” collection included taxa which were not present in the “MicroEcoEvo” collection, such as *Pseudomonas* (11 isolates), *Alteromonas* (3 isolates), *Thalassomonas* (2 isolates) and *Grimontia* (1 isolate), highlighting the taxonomic complementarity of both collections. Moreover, while *Alphaproteobacteria* (61% of all isolates) dominated the “MicroEcoEvo” collection, *Gammaproteobacteria* (86% of all isolates) dominated the “EcoTech-SPONGE” collection. The “MicroEcoEvo” collection was derived from six marine animal species and a variety of culture media and conditions, including low nutrient content media (e.g., diluted Marine Agar (MA), 50× diluted marine Gellan Gum—MG50 [25], marine R2A [34], and marine VXA [35]), and incubation temperatures of 18 °C, 19 °C or 25 °C to promote taxonomic diversity of culturable marine bacteria. Particularly MG50 and R2A provided a higher diversity of culturable genera at a low cultivation effort (≤50 isolates) compared with full strength MA. The “culturable diversity score” (i.e., the number of genera obtained over the total number of isolates gathered in a cultivation attempt) was 0.3061 for MG50 and 0.1203 for R2A but only 0.0827 for MA. In absolute numbers, however, Marine Agar (full strength and half strength (1:2 diluted)) yielded the largest number of marine host-associated bacterial genera in our collections (but also had the highest cultivation effort). While MA allowed cultivation of symbiotic taxa such as *Endozoicomonas* and *Parendozoicomonas*, the other marine media allowed the isolation of 17 genera that were not obtained on Marine Agar, such as, e.g., *Anderseniella* and *Tateyamaria* on MG50, *Lentilitoribacter*, and *Marinicauda* on R2A, and *Agarivorans* and *Alcanivorax* on VXA. The proportion of *Gammaproteobacteria* versus *Alphaproteobacteria* in the “MicroEcoEvo” collection was seemingly influenced by the nutrient content (particularly carbon and nitrogen) present in the medium. For example, in the closely related octocorals *Eunicella verrucosa* and *E. labiata* sampled off the coast of Algarve, cultivation on full strength MA at 19 °C yielded 91% *Gammaproteobacteria* (i.e., *Vibrio*, *Shewanella* and *Pseudoalteromonas*) and only 3 % *Alphaproteobacteria* isolates from *E. verrucosa* [36], while cultivation on half strength MA at a similar temperature (18 °C) yielded only 15% *Gammaproteobacteria* isolates and 80% *Alphaproteobacteria*, with *Ruegeria*, *Roseibium*, *Roseovarius* and *Sphingorhabdus* as most frequently cultured genera from *E. labiata* [27].

### 2.2. Phylogenomic Inference of Marine Host-Associated Bacteria 

**Figure 3:**
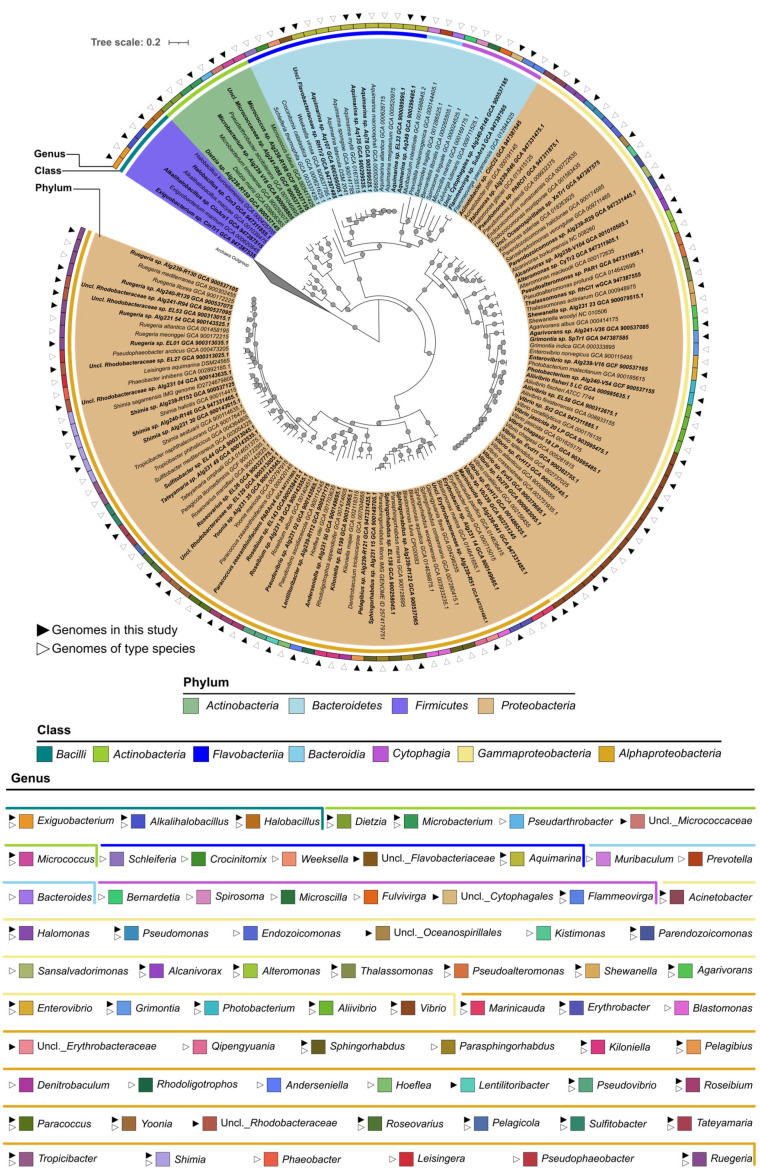
Phylogenomic inference of the 70 genome-sequenced isolates from the “MicroEcoEvo” and “EcoTechSPONGE” collections (highlighted by black triangles). In addition, 84 genomes obtained from public databases and representing type strains of species with standing in nomenclature (empty triangles) were added to the tree. Black circles on tree branches represent resampling (1000 repetitions) support greater than 75%. Five *Archaea* genomes of four type species (compressed clade) were used as an outgroup to root the tree, consisting of *Archaeoglobus fulgidus*, *Methanobacterium formicicum*, *Nitrososphaera viennensis* and *Thermoplasma acidophilum*. For each genome, corresponding species or strain names are shown, followed by the respective genome assembly accession number. Colored background shading represents the phylum-level classification of the genomes. The inner ring represents the class level taxonomy, and the outer ring represents the genus level affiliation of each genome. The legends are organized from left to right by order of appearance in the tree; on top of the “genus” legend, a colored bar indicates the bacterial class each genus belongs to. The evolutionary history was inferred by using a Maximum Likelihood method (FastTree2) and the Jukes-Cantor+CAT model based on alignment similarity of a set of 49 core, universal genes defined by COG (Clusters of Orthologous Groups) gene families. The evolutionary analyses were conducted using the SpeciesTreeBuilder v.01.0 app of DOE Systems Biology Knowledgebase (KBase). Uncl.—Unclassified.

Seventy isolates from the four phyla and six classes of both culture collections (56 from the “MicroEcoEvo” and 14 from the “EcoTech-SPONGE” collection, to harmonize between cultivation and genome sequencing efforts) had their genomes sequenced with Illumina technology, assembled, and annotated (Table S3). Fifty of these genome assemblies (all from the “MicroEcoEvo” collection) were already publicly available, while twenty genomes are original contributions of this study and reported here for the first time (Table S3). Genomes were overall of high quality, with 66/70 showing over 97% completeness and 69/70 less than 4% contamination (Table S3). Most genomes belonged to the phylum *Proteobacteria*, featuring 30 alphaproteobacterial and 25 gammaproteobacterial strains (Figure 3). The remaining 15 genomes belonged to six *Flavobacteriia*, four *Actinobacteria*, three *Bacilli* and two *Cytophagia* strains (Figure 3). The genome-sequenced isolates feature multiple understudied and underrepresented species and genera. For example, our culture collection provides the only publicly available genomes for the genera *Lentilitoribacter* (strain Alg239-R112) and *Anderseniella* (strain Alg231_50), and the third genome of the genus *Pelagibius* (strain Alg239-R121). 

To investigate the phylogenomic relatedness and taxonomic novelty of the 70 genome-sequenced bacteria, particularly of the strains not classified at genus level, a phylogenomic tree was constructed. As a reference, the genomes of 84 closely related type species (Figure 3) were included in the analysis. The closest type species of our 70 genome-sequenced strains were identified using the Microbial Genomes Atlas (MiGA) [37], based on average nucleotide identity (ANI) and average amino acid identity (AAI) scores. Most of the 70 genomes clustered with the genomes of their respective closest type strains (Figure 3). Unclassified *Flavobacteriaceae* sp. strain RHTr2 from the “EcoTech-SPONGE” collection branched next to the *Aquimarina* genus (Figure 3) and shared 66.49% AAI (close to the 65% genus-level cut-off of MiGA) with its closest type strain, *Aquimarina spongiae* DSM 22623 (T). Moreover, based on 16S rRNA gene sequencing, strain RHTr2 was affiliated to the *Aquimarina* genus with 78% confidence only (i.e., below the 80% confidence threshold of the RDP classifier tool). This, together with the phylogenomic tree topology, suggests that strain RHTr2 could represent a novel genus within the *Flavobacteriaceae* family, possessing close relatedness to the genus *Aquimarina*. Unclassified *Cytophagales* sp. strain Alg240-R148 from the “MicroEcoEvo” collection formed a clade with *Fulvivirga lutimaris* (GCA_009711525.1) on the phylogenomic tree. However, it shared only 55.33% and 55.02% AAI (much below the 65% AAI genus-level cut-off) with its closest type species, *Fulvivirga kasyanovii* JCM 16186T and *Fulvivirga lutimaris* (GCA_009711525.1), respectively. Based on 16S rRNA gene homology, the RDP classifier tool did not provide family or genus-level assignments with sufficient confidence (only 68% confidence for the family *Fulvivirgaceae*). This suggests that strain Alg240-R148 represents a novel genus and possibly even a novel family within the *Cytophagales* order, likely closely related to the *Fulvivirgaceae* family (Figure 3). 

Unclassified *Oceanospirillales* sp. strain XeTr1 (“EcoTech-SPONGE” collection) clustered among genomes of the *Endozoicomonadaceae* family, forming a sister clade to *Kistimonas asteriae* (GCA_018263925), the only publicly available genome of a *Kistimonas* isolate (Figure 3). Yet, it shared only 58.54% AAI with its closest type strain, *Endozoicomonas montiporae* (GCA_001583435), as identified by MiGA, and ~58% AAI to four other *Endozoicomonadaceae* genomes (Table S4). Moreover, strain XeTr1 was classified into the *Endozoicomonadaceae* family by the RDP classifier tool with only 63% confidence (well-below the 80% cut-off). Therefore, strain XeTr1 could represent a novel genus and possibly even a novel family closely related to the *Endozoicomonadaceae* family, and to the *Kistimonas* and *Endozoicomonas* genera.

Unclassified *Erythrobacteraceae* sp. strain Alg239-R51 (“MicroEcoEvo” collection) formed a cluster with *Qipengyuania flava* (GCA_004296255) (Figure 3) and shared >67% AAI with the genomes of six *Qipengyuania* type species (Table S4), suggesting that the strain may belong to the recently described *Erythrobacteraceae* genus *Qipengyuania*. Phylogenomics inference of the alphaproteobacterial isolates of the “MicroEcoEvo” collection further suggested re-classification of *Sphingorhabdus* strains EL138 and Alg231-15 into the genus *Parasphingorhabdus* and placement of unclassified *Rhodobacteraceae strain* EL27 into the genus *Pseudophaeobacter.* A more detailed description of the phylogenomics results for the alphaproteobacterial isolates is provided in the ‘Extended Results’ section of Supplementary File S1. 

To further assess the functional and phylogenomic relatedness among the 70 genome-sequenced strains, a multivariate analysis using protein family (Pfam) annotations of the 70 genomes was conducted and is also described in the ‘Extended Results’ section of Supplementary File S1. The principal coordinates analysis easily distinguished among bacterial classes (Figure S1, Table S6), showing that, overall, functional gene profiles recapitulate phylogeny.

### 2.3. Identification of Secondary Metabolite Biosynthetic Gene Clusters (SM-BGCS) in Marine Bacterial Genomes 

**Figure 4:**
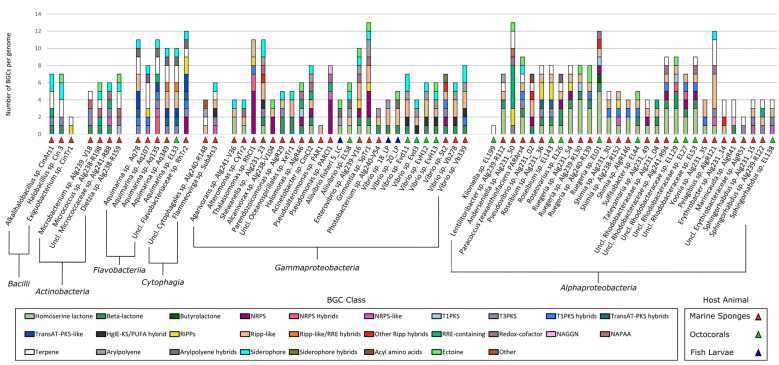
Secondary metabolite biosynthetic gene cluster (SM-BGC) counts for each of the 70 genome-sequenced marine bacterial isolates. SM-BGCs were grouped into 28 compound classes as shown in the legend. The order (left to right) in the legend corresponds to the order of appearance in the stacked bar chart (bottom to top). Original antiSMASH classification results are available in Table S7. The class-level taxonomic affiliation of isolates is indicated below the strain names. The host origin of each isolate is represented by a colored triangle next to the strain name: red—marine sponges, green—octocorals and blue—seabream larvae. NAGGN—N-acetylglutaminylglutamine amide; NAPAA—non-alpha poly-amino acids like e-polylysin; NRPS—non-ribosomal peptide synthetase; PKS—polyketide synthase; RiPP—ribosomally synthesized and post-translationally modified peptides. Uncl.—unclassified.

Using antiSMASH v6.0.0 [19], 466 SM-BGCs of 28 compound classes were identified in the 70 genomes (Figure 4). All details of the antiSMASH classification, including SM-BGC size and location in the genome are provided in Table S7. Overall, the number of SM-BGCs per genome varied from one SM-BGC in strains *Kiloniella* sp. EL199 and *Pseudoalteromonas* sp. PAR1 to 13 SM-BGCs in strains *Grimontia* sp. SpTr1 and *Anderseniella* sp. Alg231_50. Twelve isolates possessed more than 10 SM-BGCs, while the average SM-BGC count was 6.5 SM-BGCs per genome (Figure 4). Of the 466 SM-BGCs, 38 had above 70% gene cluster similarity with SM-BGCs that produce known compounds and that are present in the MIBiG database (Table S8). Of those 38 SM-BGCs, 20 coded for carotenoids, 11 for iron chelating compounds (i.e., bisucaberin B, aerobactin, acinetobactin, desferrioxamine E and amphi-enterobactins 1/2/3/4) and seven for ectoine, known to be involved in osmoregulation (Table S8).

The *Bacilli* isolates *Alkalihalobacillus* sp. CinArs1 and *Halobacillus* sp. Cin3 shared similar SM-BGC profiles, each harboring a type III polyketide synthase (T3PKS) and a betalactone cluster, while *Exiguobacterium* sp. CinTr1 harbored one ribosomally synthesized and post-translationally modified peptide (RiPP) SM-BGC (Figure 4). Likewise, *Actinobacteria* isolates *Microbacterium* sp. Alg239_V18 and unclassified *Micrococcaceae* sp. Alg241-R88 harbored each a T3PKS cluster, while *Dietzia* sp. Alg238-R159 harbored a type I polyketide synthase (T1PKS) cluster (Figure 4).

The *Flavobacteriia* isolates investigated in this study showed quite unique SM-BGC profiles, with *Aquimarina* sp. strains Aq78, Aq349 and EL33 hosting many PKS SM-BGCs, as well as RiPP-like clusters (Figure 4). Unclassified *Flavobacteriaceae* sp. strain RHTr2 harbored five PKS SM-BGCs (including T1PKS, T3PKS and transAT-PKS), in addition to two NRPS and two RiPP clusters (Figure 4), pointing towards a very rich and novel secondary metabolism in this potentially novel taxon.

None of the *Gammaproteobacteria* genomes harbored terpene SM-BGCs, which were present in all other bacterial classes investigated here (Figure 4). Overall, the *Gammaproteobacteria* SM-BGC content was highly diverse. The SM-BGC profile of the marine sponge derived *Grimontia* sp. SpTr1 genome showed similarities to that of sponge-derived *Enterovibrio* sp. strain Alg239-V16; both harbored beta-lactone, NRPS, RiPP and RiPP-like clusters (Figure 4). *Thalassomonas* sp. strain RHCl1 stood out with five NRPS clusters (Figure 4).

The *Alphaproteobacteria* strains also displayed a varied secondary metabolite biosynthesis potential, but most strains lacked siderophore clusters (Figure 4). The *Rhodobacteraceae* family was enriched in homoserine lactone SM-BGCs. Octocoral-derived *Ruegeria* sp. EL01 and the unclassified *Rhodobacteraceae* sp. strains EL27, EL53, EL129 and sponge-derived Alg241-R94 all harbored, among others, one NRPS, one RiPP-like and one T1PKS or T1PKS hybrid cluster in their genomes (Figure 4). Marine sponge-derived *Pelagibius* sp. strain Alg239-R121 and *Anderseniella* sp. strain Alg231_50 stood out for their many RiPP-like clusters (Figure 4).

### 2.4. Network Analysis of SM-BGCs 

**Figure 5:**
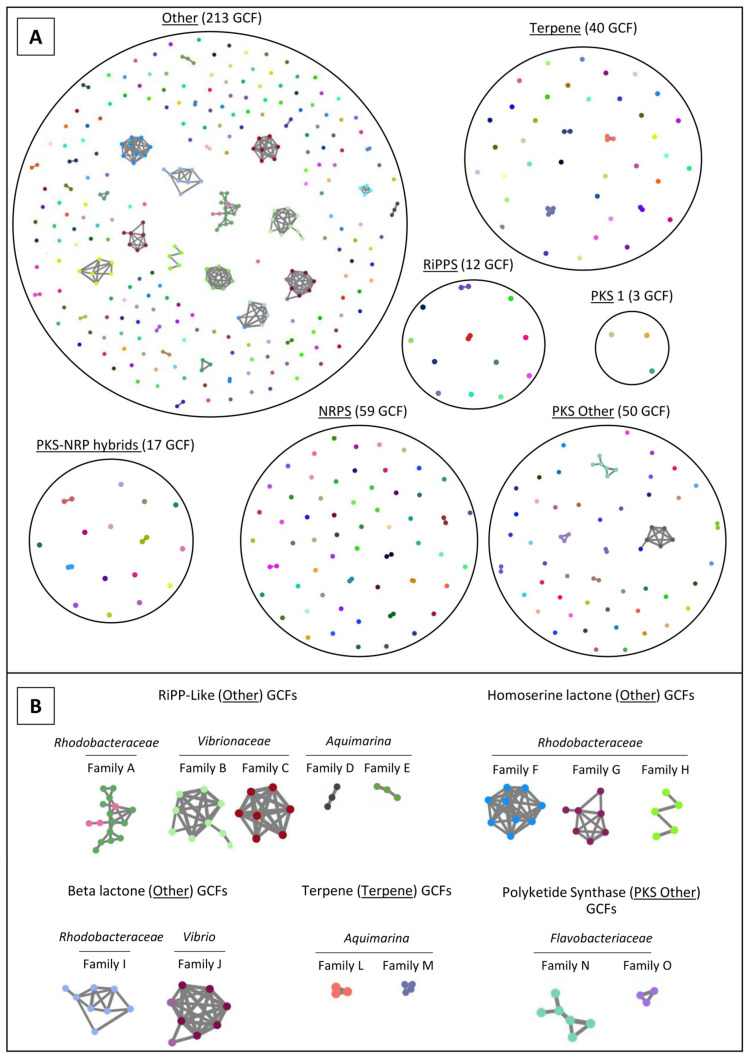
Sequence similarity network analysis of the SM-BGCs from the 70 genome-sequenced marine bacterial isolates. Each colored node represents a single SM-BGC identified by antiSMASH v6. Grey lines connecting certain SM-BGCs indicate their respective grouping into Gene Cluster families (GCFs). There were 337 singleton-GFCs (i.e., SM-BGCs with no relevant similarity to other SM-BGCs) and 57 GCFs that consisted of two or more SM-BGCs in the dataset. (**A**) Organization of SM-BGCs and GCFs according to BiG-SCAPE’s compound class nomenclature. (**B**) Taxon-specific GCFs identified among the different compound classes. A list of the bacterial isolates belonging to each taxon-specific GCF is available in Table S9.

To determine the similarity of the 466 SM-BGCs annotated across the 70 marine bacterial genomes, similarity networks were generated using BiG-SCAPE v1 [38]. In total, the 466 SM-BGCs grouped into 394 gene cluster families (GCFs), i.e., groups of SM-BGCs that share >70% similarity according to a BiG-SCAPE metric which integrates number of shared Pfam domains, synteny of the Pfam domains and sequence similarity. Fifty-seven GCFs consisted of two or more SM-BGCs. The remaining 337 GCFs (85%) were singletons, i.e., SM-BGCs with no relevant similarity to other SM-BGCs (Figure 5A). Many SM-BGCs annotated by antiSMASH v.6.0.0 were not classified under the BiG-SCAPE nomenclature, and instead grouped under the category “Other”. 

Several large GCFs, consisting of SM-BGCs of phylogenetically related bacteria, were present in the compound categories “Other”, “Terpene” and “PKS Other” (Figure 5A). The GCFs A–E (Figure 5B) were classified as “RiPP-like” by antiSMASH, which is a broad term for SM-BGCs similar to RiPPs [19]. RiPP-like family A consisted of SM-BGCs from 16 of the 17 *Rhodobacteraceae* (all except *Paracoccus zeaxanthinifaciens* strain ParArs4) genomes of this study (Table S9). The GCFs F–H encompassed homoserine lactone SM-BGCs classified by antiSMASH. These GCFs consisted solely of SM-BGCs from isolates of the *Rhodobacteraceae* family (Figure 5B). Each of these GCFs (F, G, H) consisted of SM-BGCs from at least two *Rhodobacteraceae* genera, although SM-BGCs from one genus dominated each GCF (Table S9). Notably, many *Rhodobacteraceae* isolates (12 out of 16 strains) that harbored a RiPP-like SM-BGC of GCF A also had one or more homoserine lactone SM-BGCs in GCFs F, G and/or H (Figure 5B, Table S9). The genomes to which the SM-BGCs of GCFs A–O belong are detailed in Table S9.

### 2.5. Antimicrobial Activities of Marine Bacterial Isolates

Following the detailed inspection of their secondary metabolite biosynthesis capacities, the genome-sequenced isolates were screened for antifungal and antibacterial activities against five human-pathogenic fungal strains, *Candida glabrata* KCHR606 [39] and Δpdr1 deletion mutant [40] (the latter with a full knockout of the *PDR1* gene, encoding for the master regulator of azole drug resistance in *C. glabrata)*, *C. albicans* ATCC SC5314, *C. parapsilosis* ATCC 22019 and *C. auris* ATCC B11220, and four human-pathogenic bacteria, *Staphylococcus aureus* ATCC 6538, methicillin-resistant *Staphylococcus aureus* (MRSA) strain JE2 (NR-46543), *Salmonella enterica* subsp. *enterica* serovar Typhimurium DSM 24522 and *Vibrio parahaemolyticus* DSM 101031 (the latter being a human, fish and shellfish pathogen).

**Figure 6:**
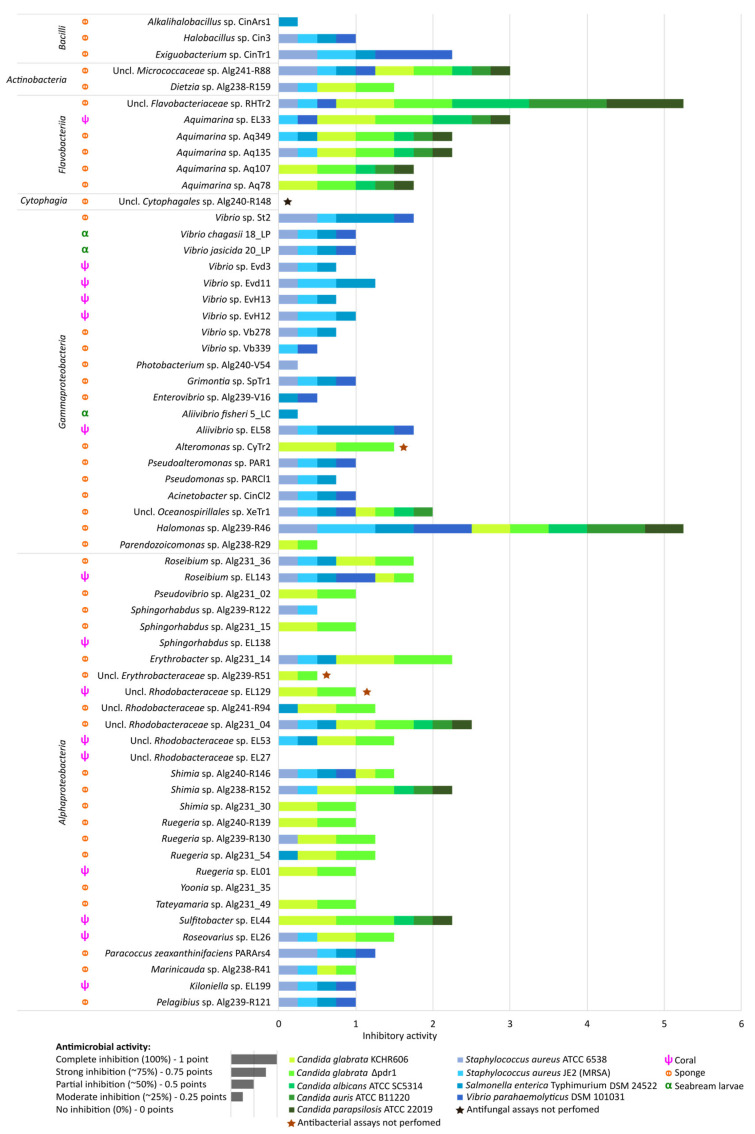
Antimicrobial activities of the marine bacterial isolates against human-pathogenic bacteria and *Candida* spp. strains. Cross-streak agar plate assay results are shown. Inhibition of pathogen growth in the presence of marine bacterial isolates was assessed qualitatively in a screening-based fashion. The stacked bar size indicates the intensity of observed growth reduction compared with negative controls (i.e., pathogens grown without the presence of marine bacteria). The antimicrobial activities were ranked on a 0—1 scale as follows: No pathogen growth inhibition—0 points; Weak inhibition—0.25 of a point; Moderate inhibition—0.50 of a point; Strong inhibition–0.75 of a point; Complete inhibition of pathogen growth–1.0 point. Different colors represent the various pathogens tested, whereby shades of green represent *Candida* spp. and shades of blue represent bacterial pathogens. Uncl.—unclassified.

Of the 70 genome-sequenced isolates, nine strains did not grow well enough in the cross-streak assays [41] used here for antimicrobial activity screenings. Test results are, therefore, presented for 61 genome-sequenced bacteria. Of those 61 bacteria, 57 showed some degree of activity against at least one pathogen (Figure 6). Among the *Candida* pathogens, *C. glabrata* was the most susceptible species, with 33 marine bacterial isolates causing some degree of growth inhibition in *C. glabrata*, irrespective of the presence or absence of the *PDR1* gene. Thirteen of the 33 isolates were able to reduce growth of all *Candida* strains tested (Figure 6). We also observed growth inhibition of Gram-positive and Gram-negative human-pathogenic bacteria by marine isolates, with 35 isolates active against *S. aureus* ATCC 6538, 37 against MRSA strain JE2 (NR-46543) and 33 against *S. enterica* serovar Typhimurium DSM 24522 (Figure 6). *Vibrio parahaemolyticus* DSM 101031 was the least susceptible species in this study; only 21 marine bacterial isolates inhibited its growth. 

While none of the *Bacilli* isolates showed antifungal activity, *Halobacillus* sp. CinArs1 and *Exiguobacterium* sp. CinTr1 showed activity against all bacterial pathogens tested here (Figure 6). Of the marine *Actinobacteria*, unclassified *Micrococcaceae* sp. Alg241-R88 showed weak activity against all tested pathogens. The six marine *Flavobacteriaceae* strains tested in this study showed relatively little antibacterial activity in the cross-streak assays but notable antifungal activity (Figure 6). Marine sponge derived unclassified *Flavobacteriaceae* sp. strain RHTr2 showed strong antifungal activity and was the only isolate which completely inhibited *C. albicans*, *C. auris* and *C. parapsilosis* (Figure 6). 

Most (19 out of 21) marine gammaproteobacterial isolates showed some activity against pathogenic bacteria, while only four gammaproteobacterial isolates, of the orders *Oceanospirillales* and *Alteromonadales*, inhibited any of the *Candida* pathogens (Figure 6). *Oceanospirillales* isolate *Halomonas* sp. Alg239-R46 showed strong activity against bacterial and fungal pathogens, while unclassified *Oceanospirillales* sp. strain, XeTr1 was weakly active against all tested bacterial and fungal pathogens except for *C. parapsilosis* (Figure 6). Five strains of the *Vibrionaceae* family inhibited the four bacterial pathogens, whereby sponge-derived *Vibrio* sp. St2 and octocoral-derived *Aliivibrio* sp. EL58 showed strong activity against *S. enterica* serovar Typhimurium. However, none of the 14 tested *Vibrionaceae* strains, spanning five genera (i.e., *Vibrio*, *Aliivibrio*, *Enterovibrio*, *Photobacterium* and *Grimontia*), showed antifungal activity (Figure 6). 

The marine alphaproteobacterial isolates were generally more active against fungal than bacterial pathogens (Figure 6). Most strains inhibited the growth of *C. glabrata* (21 out of 28), with unclassified *Rhodobacteraceae* sp. Alg231_04, *Shimia* sp. Alg238-R152 and *Sulfitobacter* sp. EL44 also inhibiting *C*. *albicans*, *C. auris* and *C. parapsilosis* growth (Figure 6). Five alphaproteobacterial isolates, *Roseibium* sp. EL143, *Shimia* sp. Alg240-R146, *Paracoccus zeaxanthinifaciens* PARArs4, *Kiloniella* sp. EL199 and *Pelagibius* sp. Alg239-R121, inhibited the four bacterial pathogens to some extent (Figure 6). Overall, the type of host animal (i.e., marine sponge, octocoral or fish) was not a determining factor for antimicrobial activity since activities were observed in isolates from the three host types. Nevertheless, two marine bacterial strains that truly stood out in this study for their strong and broad-spectrum antimicrobial activities, *Halomonas* sp. Alg239-R46 and *Flavobacteriaceae* sp. RHTr2, were both derived from marine sponges (*Spongia officinalis* and *Rhabdastrella globostellata*, respectively).

## 3. Discussion

### 3.1. The “MicroEcoEvo” and “EcoTech-SPONGE” Culture Collections Feature a Series of Underexplored and Taxonomically Novel Marine Bacteria

This study explored the genetic diversity of 919 marine host-associated bacterial isolates from two culture collections, altogether encompassing four phyla, six classes, 17 orders, 33 families and 55 described genera, in addition to 30 isolates that were unclassifiable at either genus (19 strains), family (7 strains), or even order (4 strains) level, and most likely represent novel taxa. Unclassified *Flavobacteriaceae* sp. strain RHTr2 (phylum *Bacteroidetes*) showed strong antimicrobial activity in this study and was closely related to *Aquimarina*, a marine *Flavobacteriaceae* genus, which is receiving increased attention for its versatile secondary metabolism [42,43], antimicrobial activities [43], and as a source of novel polyketides and antibiotic peptides [44,45]. However, neither whole genome-based AAI values, nor 16S rRNA gene similarity, offered strong enough support to classify RHTr2 as *Aquimarina*. Interestingly, the 16S rRNA gene sequence of an uncultured *Flavobacteriaceae* bacterium [46] from the coast of Guam (Pacific Ocean) and associated with the marine sponge *Rhabdastrella globostellata*, the same sponge species from which RHTr2 was obtained, shared 99.64% similarity with isolate RHTr2. It could suggest that this novel taxon may favor associations with marine sponges. 

The unclassified *Micrococcaceae* strain Alg241-R88 (phylum *Actinobacteria*) of the “MicroEcoEvo” collection, was one of only two isolates in this study that showed inhibitory activity against all tested pathogens. Phylogenomically close to the genus *Pseudarthrobacter*, a sibling of the *Arthrobacter* genus, strain Alg241-R88 possessed a NRPS-like and a T3PKS SM-BGC. *Arthrobacter* spp. have been reported to possess antifungal and antibacterial [47] activity. Relatively little is known about the genus *Pseudarthrobacter*, yet two *Pseudarthrobacter oxydans* strains showed activity against *S. aureus* and *C. albicans* in a recent study [48].

The “EcoTech-SPONGE” collection harbored six unclassified *Oceanospirillales* isolates which likely belong to a novel, not yet described family, closely related to the *Endozoicomonadaceae* family. The halophilic and metabolically versatile order *Oceanospirillales* not only contains many endosymbiotic taxa [49], but also polyester- and oil-degrading groups, as well as deep-water dwellers [50]. Our *Oceanospirillales* sp. strain XeTr1 from *Xestospongia* sp. showed weak antimicrobial activity against all tested microbial pathogens, except for *C. parapsilosis*, and its SM-BGC profile was characterized by beta-lactones, a compound class known for its chemical diversity and clinical potential [51]. 

### 3.2. Both Culture Collections Comprise Taxa Typically Known for Their Symbiotic Lifestyles

Seven strains in this study, one of them with a sequenced genome, belonged to the family *Endozoicomonadaceae* (*Oceanospirillales, Gammaproteobacteria*), a family typically found in association with marine animals and formally described only in 2018 [52]. Indeed, the core microbiome of corals, particularly of temperate octocorals, primarily consists of *Gammaproteobacteria*, with *Endozoicomonadaceae* phylotypes frequently dominating the prokaryotic community [18,21]. Members of this family have been suggested to be involved in sulfur and carbon cycling in their hosts [53,54] and are indicators of coral health [21]. Moreover, metagenomic analyses have suggested a role for *Endozoicomonadaceae* symbionts in the processing of chitin, the most abundant natural polysaccharide in marine environments and a major component of the zoo- and phytoplankton prey of suspension-feeding marine invertebrates [54,55]. In a recent study, unculturable, symbiotic *Endozoicomonadaceae* lineages did not possess any SM-BGCs in their genomes, suggesting that their main function is not chemical defense but rather nutrient provision and cycling [54]. Some cultured *Endozoicomonadaceae* representatives, however, were found by Sweet et al. to possess bacteriocin (RiPP) clusters [56]. *Parendozoicomonas* sp. Alg238-R29 was revealed in this study to also harbor a T3PKS cluster in addition to the RiPP cluster, although its antimicrobial activity in the cross-streak assays was negligible. The halophilic genus *Halomonas (Halomonadaceae, Oceanospirillales)* has been used in probiotic treatments of shrimp due to its antiviral and anti-microbial activities [57,58] and was also part of a ‘Beneficial Microbes for Corals’ (BMC) consortium that prevented corals from bleaching under pathogen and temperature stress [59]. Both the coral-associated *Halomonas* BMC strain [56] and our marine sponge-derived *Halomonas* sp. Alg239-R46 harbored a T1PKS cluster in their genomes. Indeed, *Halomonas* sp. Alg239-R46 was among the most bioactive isolates of this study, showing inhibitory activity against all bacterial and fungal pathogens tested and strong inhibition against MRSA, *V. parahaemolyticus* and *C. auris*. These results suggest competitive ability of *Halomonas* spp. in host-associated settings through the release of antimicrobial compounds, which could lead to chemical defense of their natural hosts against fungal and bacterial infections.

The marine bacterial genus *Pseudoalteromonas* (*Pseudoalteromonadaceae*, *Alteromonadales*) is another taxon frequently found in association with eukaryotic hosts [56,60,61] and several *Pseudoalteromonas* strains were among the above-mentioned BMC consortium [59]. Our two culture collections consisted of 28 *Pseudoalteromonas* strains, 26 isolated from marine sponges and two from the octocoral *Eunicella verrucosa*. *Pseudoalteromonas* spp. are versatile antimicrobial compound producers, with 69 antimicrobial compounds reported until 2016 [61]. More recently, a clade consisting of at least 19 *Pseudoalteromonas* members was reported by Chau and colleagues to possess large numbers of SM-BGCs (on average >10 SM-BGCs per genome), including SM-BGCs which produce polyketides, non-ribosomal peptides and RiPPs/bacteriocins [62]. In this study, *Pseudoalteromonas* sp. PARCl1 displayed moderate antibacterial activity and was also found to harbor multiple NRPS SM-BGCs. However, strain PARC11 is phylogenetically different from the SM-BGC-enriched *Pseudoalteromonas* clade described by Chau and colleagues [62]. In contrast, 14 other *Pseudoalteromonas* sp. strains of the “EcoTech-SPONGE” collection had >99% 16S rRNA gene similarity to the abovementioned SM-BGC-enriched *Pseudoalteromonas* [62] (Table S10). These 14 *Pseudoalteromonas* strains may thus be a valuable target for future bioprospection studies, possibly also displaying a complex secondary metabolism.

Finally, *Roseibium* (homotypic synonym *Labrenzia*) is a marine alphaproteobacterial genus (family *Stappiaceae*, order *Hyphomicrobiales*) that is frequently isolated from filter- or suspension-feeding, sessile invertebrates such as sponges [25], corals [27,63], and oysters [64]. Several compounds have already been isolated from this genus, including labrenzbactin, a siderophore with antibacterial and anticancer activity [65], and labrenzin, an analogue of the polyketide pederin with anticancer activity [66]. The “MicroEcoEvo” collection harbored as many as 62 *Roseibium* strains and, in this study, *Roseibium album* strains Alg231_36 and EL143 showed inhibitory activity towards human-pathogenic bacteria and *C. glabrata.* In line with earlier findings for the genus [67], both strains harbored transAT-PKS, T3PKS, NRPS and multiple RiPP-like SM-BGCs, underpinning their potential to produce a diverse range of secondary metabolites.

### 3.3. Reduction of Carbon-Content and Prolonged Incubation Periods Promote an Increased Diversity of Culturable Marine Bacteria

The cultivation of sponge-associated bacteria on a range of culture media with varying carbon sources and concentrations and additives has been used as a strategy to expand the repertoire of culturable sponge- and coral-associated bacteria [56,60]. It has been suggested that bacterial division rates within the sponge microbiome are relatively low, wherefore slow-growing bacteria may only be captured with long cultivation times [60]. Moreover, cultivation is a competitive process and high carbon-content media tend to favor fast growing genera (r-strategists) such, e.g., *Vibrio* and *Pseudovibrio*, allowing them to develop colonies quickly. Within 2–3 days of incubation, they may take over the entire surface of the cultivation plate and can prevent slow-growing taxa (k-strategists) to emerge, as observed elsewhere [24]. The *Spongia officinalis*-associated bacteria of the “MicroEcoEvo” collection were cultured on three low-carb media, R2A [34], MG50 [25] and VXA [35] (instead of nutrient-rich MA) with an incubation period of eight weeks, which promoted the growth of 15 bacterial families that were not obtained on full strength marine agar. These conditions likely contributed to the cultivation of slow-growing or difficult-to-cultivate marine taxa, such as *Anderseniella*, *Lentilitoribacter*, *Pelagibius*, *Tateyamaria*, *Marinicauda* and *Parendozoicomonas*, all understudied genera with limited numbers of known species and genomes available. Indeed, according to the List of Prokaryotic Names with Standing in Nomenclature (LPSN) [68] (last checked September 2022), the genera *Lentilitoribacter, Anderseniella* and *Parendozoicomonas* comprise only one formally described species each, while *Pelagibius* consists of two species and *Tateyamaria* and *Marinicauda* three species, respectively. Likewise, according to NCBI [69] (last checked September 2022), very few genomes are so-far available from cultured strains of these genera: seven for *Tateyamaria* and *Marinicauda* each, three for *Pelagibius*, two for *Parendozoicomonas*, and one for *Anderseniella* and *Lentilitoribacter*, respectively, including the genomes described in this study. This not only highlights the suitability of low-carb media and long incubation periods for the cultivation of understudied marine bacteria, but also emphasizes the important contribution of the here described culture collections as a resource of novel marine bacterial strains and genomes of understudied lineages.

### 3.4. The “MicroEcoEvo” and “EcoTech-SPONGE” Collections Are a Reservoir of Chemical Novelty with Most SM-BGCs Showing Little Homology to Those of Known Compounds

This study identified 466 SM-BGCs across 70 marine bacterial genomes, with 428 SM-BGCs (>90%) having little or no homology to SM-BGCs coding for known compounds. This suggests a vastly underexplored chemical diversity among our culture collections. RiPP-like, NRPS, PKS and terpene SM-BGCs were detected most frequently, all of which are compound classes that have been associated with antimicrobial molecule production in the past. The 38 SM-BGCs with high homology (>70%) to SM-BGCs of known compounds included those producing molecules that are pigments and/or have antioxidant functions (e.g., carotenoids, flexirubins), and those involved in osmoregulation (ectoine), and iron sequestration (e.g., bisucaberin B, aerobactin). These compounds may provide important ecological advantages to marine bacteria in their natural habitats, and are among the factors which support beneficial interactions with marine animal hosts [70]. 

The *Flavobacteriaceae* isolates examined in this study stood out for their many polyketide-synthase encoding SM-BGCs, e.g., T1PKS, T3PKS, transAT-PKS and hybrid-PKS SM-BGCs, with six of the transAT-PKS and three of the T3PKS forming GCFs N and O, respectively. TransAT-PKS GCF N was present in the genomes of *Aquimarina* sp. strains Aq78, Aq349 and EL33 as well as unclassified *Flavobacteriaceae* strain RHTr2, and corresponded to the recently identified polyketide cuniculene [44]. Silva et al. [43] demonstrated that the cuniculene SM-BGC is shared by several *Aquimarina* species, and the present study shows that other members of the *Flavobacteriaceae* family may also possess the capacity to produce cuniculene or derivatives thereof. The ecological role and bioactivity of cuniculene is yet to be described [44]. However, other natural products synthesized by transAT-PKSs exhibit antibiotic [71] and anticancer [72] activities, among others. The SM-BGCs belonging to T3PKS family O, present in the genomes of *Aquimarina* strains Aq78, Aq349 and EL33, were not like any SM-BGC of a known compound present in the MIBiG database, and most likely encode for a novel polyketide. These three *Aquimarina* strains indeed share similar metabolomes, although most of their secondary metabolites have not yet been identified [43]. 

Of the 25 *Gammaproteobacteria* genomes analyzed in this study, four isolates stood out for their large number (10–13) of SM-BGCs. One of them was *Shewanella* sp. strain Alg231_23, characterized by several RiPP clusters in addition to a T3PKS cluster. Two macrocyclic polyketides with antibacterial activity against several multi-resistant bacteria, including MRSA, were recently identified in *Shewanella algae* [73]. The “MicroEcoEvo” collection hosted as many as 25 *Shewanella* (*Shewanellaceae*, *Alteromonadales*) strains, obtained from the octocorals *E. labiata* and *E. verrucosa* and marine sponges *S. spinosulus* and *I. variabilis*. *Shewanella* spp. are common members of the microbiome of algae [74], molluscs [75] and sponges [76]. Some strains are known to have probiotic effects on *Sparus aurata* and *Solea senegalensis*, reducing larval and/or juvenile mortality [77] as they increase host resistance to pathogens [78]. The secondary metabolisms of the other three gammaproteobacterial genera with ≥10 SM-BGCs, namely *Enterovibrio* sp. Alg239-V16, *Grimontia* sp. SpTr1 and *Thalassomonas* sp. RhCl1, notable for their many NRPS and RiPP clusters, are still vastly underexplored and literature reports on the topic are scarce. Nevertheless, one *Thalassomonas*-derived RiPP cluster was recently characterized and its corresponding lanthipeptides, thalassomonasin A and B, were produced by heterologous expression in *Escherichia coli* [79]. 

RiPP BGCs from 16 of the 17 *Rhodobacteraceae* (*Alphaproteobacteria*) isolates (all except *Paracoccus zeaxanthinifaciens* PARArs4) analyzed in this study grouped together in one gene cluster family (A), given their high similarity. RiPP compounds are often antibacterial agents, particularly the bacteriocin subclass of RiPPs is widely known for its antibacterial activity [80]. However, peptides of this SM-BGC class can have many functions, some acting as siderophores, while others are involved in biofilm formation and maintenance [81]. All *Rhodobacteraceae* genomes included at least one N-acyl homoserine lactone (AHL) SM-BGCs, with all *Ruegeria* and four of the unclassified *Rhodobacteraceae* strains hosted two to five AHL BGCs per genome. AHLs are involved in chemical communication such as quorum sensing, a mechanism which, among others, frequently controls secondary metabolite production in *Proteobacteria*. Martens et al. (2007) found that the presence of PKS and NRPS genes in marine *Rhodobacteraceae* correlates to some extent with AHL production and antagonistic activity [82]. Moreover, the synthesis of the antimicrobial compound tropodithietic acid (TDA) in *Phaeobacter* and *Ruegeria* spp. is mediated by AHLs and quorum sensing [83]. Most *Rhodobacteraceae*-derived AHL SM-BGCs reported in this study belonged to one of three GCFs. Notably, in strains hosting multiple AHL SM-BGCs on their genomes (such as, e.g., our *Ruegeria* spp. strains), these SM-BGCs were not identical to one another but belonged to distinct AHL GCFs, suggesting that each AHL could have a distinct structure and possibly control the expression of a distinct gene set. It is not uncommon for marine *Rhodobacteraceae* genomes to possess multiple copies of luxR and/or luxI homologs, the genes which encode the proteins that produce and respond to AHLs [83]. The larger number and diversity of AHL clusters likely contributes to the structural variety of AHLs in marine *Rhodobacteraceae* strains, such as the high variation in the acyl side chains, with lengths ranging from C4 to C18 and differing degrees of saturation [83]. In our study, not every AHL SM-BGC carried a LuxR or LuxR-like domain, but all possessed an autoinducer synthase domain, indicating capacity to produce AHL-like molecules. The lack of LuxR domains in certain AHL SM-BGCs may suggest that other control mechanisms are present in our *Rhodobacteraceae* strains, as Pfam domains of unknown function were also common in these SM-BGCs.

### 3.5. Taxon-Specific Differences in Antimicrobial Activity of Marine Bacteria Suggest Distinct Mechanisms Involved in Antagonism against Human-Pathogenic Bacteria versus Candida

The present study showed that underexplored marine bacterial taxa have antimicrobial activities against canonical human pathogens, which warrants further investigation. Among the tested *Candida* pathogens, growth reduction in the presence of marine bacteria was most frequently observed in *C. glabrata*, with no difference in the susceptibility of the *Δpdr1* deletion mutant *versus* the wild type. The *PDR1* gene in *C. glabrata* encodes a transcription factor, which, in turn, induces expression of drug efflux pumps and is known to be a centerpiece in resistance to fluconazole-type antifungals [84]. Given the similar susceptibility of *C. glabrata* wild type and *Δpdr1*, the mechanisms behind the antifungal activities observed in this study are likely independent of *PDR1*-controlled resistance. This observation suggests that the underlying antifungal agents are likely to be effective against azole resistant clinical isolates. Noteworthily, *C. glabrata* appears to be more susceptible to most tested marine bacteria than the other three *Candida* species tested here, although *C. glabrata* is, of the four species, most often the one responsible for resistance to antifungal drugs, making it a particularly hard to treat pathogen [85]. Inhibition of *C. albicans*, *C. parapsilosis* and *C. auris* was less common in this study, but when it occurred, *C. glabrata* was also inhibited, indicating that the mode of action of the putative compounds involved in these cases were broad and not *Candida* species-specific. In contrast, cases where only *C. glabrata* was inhibited could suggest that the other *Candida* species share a common resistance mechanism, which *C. glabrata* lacks. Future studies should focus on the isolation and purification of the antifungal compounds to shed light on the targets for drug action. 

The Gram-positive *Exiguobacterium* sp. strain CinTr1 (*Bacillaceae*, *Firmicutes*) inhibited the growth of all bacterial pathogens and had a particularly strong effect on the fish pathogen *Vibrio parahaemolyticus*. However, there was no inhibitory activity towards *Candida*, suggesting that activities are specific for prokaryotic cells, making this strain a good candidate for further antibiotic compound research. In line with our findings, non-marine *Exiguobacterium* spp. strains are also known for their potent antibacterial activities against the human-pathogenic, Gram-negative species *Escherichia coli*, *Shigella flexneri*, *Klebsiella pneumonia* and *Salmonella enterica* [86]. 

The 14 *Vibrionaceae* strains (spanning five genera) examined in the present study showed no observable antifungal activity, but all strains showed antibacterial activity, with five of the 14 isolates inhibiting all bacterial pathogens tested here. Notably, all *Vibrionaceae* strains but *Aliivibrio fisheri* 5_LC (which had only very weak antibacterial activity) harbored one or more RiPP-like SM-BGC(s), which could serve as starting point for further investigation into the antibacterial compound(s) involved. In contrast, many alphaproteobacterial isolates, particularly members of the *Rhodobacteraceae* family, performed well against *Candida*, but poorly against bacterial pathogens, hinting at different mechanisms and molecules involved in antagonism against bacterial and fungal disease agents. *Sulfitobacter* sp. EL44, *Shimia* sp. Alg238-R152 and unclassified *Rhodobacteraceae* Alg231_04 from the “MicroEcoEvo” collection stood out among the 17 *Rhodobacteraceae* strains surveyed in this study, and showed inhibitory effects against *C. albicans*, *C. parapsilosis* and *C. auris*, in addition to *C. glabrata*. However, with four to five SM-BGCs from four to five different compound classes per genome, the above-mentioned isolates were not among the *Rhodobacteraceae* strains with the highest number and diversity of SM-BGCs (*Ruegeria* sp. EL01 and *Pelagibius* sp. Alg239-R121 had 12 SM-BGCs and 7-8 compound classes per genome, for example). This shows that the number and diversity of SM-BGCs is not necessarily a reliable predictor of in vitro bioactivity. It is not unusual for SM-BGCs to be ‘silent’ under standard culture conditions in the laboratory, i.e., the genes are not transcribed and, therefore, the underlying metabolites are not produced. Testing different experimental conditions is one way of stimulating their expression, a strategy explored by the ‘one strain, many compounds’ (OSMAC) approach [87]. Even simple changes such as switching from solid to liquid medium can already result in the biosynthesis of different metabolites, and in different inhibitory activities. This was recently shown for several *Aquimarina* spp. strains, which exhibited inhibitory activity against *S. aureus* ATCC 6538 and MRSA in broth microdilution assays with liquid culture extracts, but not in the cross-streak assays where agar plates were used [43]. A future strategy could therefore be the application of alternative antimicrobial activity screening methods, and the OSMAC approach on those strains which revealed a rich SM-BGC profile but little antimicrobial activity in the cross-streak assays.

## 4. Materials and Methods

### 4.1. The “MicroEcoEvo” and “EcoTech-SPONGE” Culture Collections

This study was embedded in the national research project “SymbioReactor”, supported by the ‘Portuguese Ministry of the Sea’, and aimed to exploit the diversity, antimicrobial activities, and natural product biosynthesis capacities of two comprehensive culture collections, totaling 919 marine host-associated bacteria. The first “MicroEcoEvo” collection comprised 800 isolates and was established between 2010 and 2020 during multiple sampling events of the temperate marine sponges *Sarcotragus spinosulus*, *Icrcina variabilis* and *Spongia officinalis* [24,25,26], the octocorals *Eunicella labiata* [27] and *Eunicella verrucosa* [36] and fish larvae of *Sparus aurata* [28,29,88] from Portugal. Sampling and sample processing were previously described [24,25,26,27,28,29,88]. Animal tissue was washed with artificial seawater to remove external bacterial colonizers after which the soft tissue was homogenized in sterile Ca_2_^+^ and Mg_2_^+^-free artificial seawater (CMFASW: 27 g L^−1^ NaCl, 1 g L^−1^ Na_2_SO_4_, 0.8 g L^−1^ KCl and 0.18 g L^−1^ NaHCO_3_, 1 g of soft tissue per 9 mL CMFASW w/v) using a sterile mortar and pestle. One milliliter of each homogenate was then serially diluted with CMFASW, and various dilutions were plated for cultivation. Several marine culture media and conditions were employed, including full strength Marine Agar [24,28,29,36,88] and half strength (1:2 diluted) Marine Agar (MA) [27], 50× diluted Marine Gellan Gum (MG50) [25] as well as R2A and VXA [26] media. Incubation temperatures ranged from 18 °C [27] to 25 °C [24] and incubation time from three days [24] to up to eight weeks [25] in the different studies (see Table S1 for more details on the isolates and culture conditions of the “MicroEcoEvo” collection). 

The second, “EcoTech-SPONGE” collection consisted of 119 bacterial isolates from 15 tropical marine sponge species of eight orders of the *Demospongia* class collected off the coast of Taiwan in 2017 [31,32]. Here, half strength (1:2 diluted) MA medium was used at 17 °C incubation temperature for cultivation of the sponge symbionts (see Table S2 for more details on the isolates and culture conditions of the “EcoTech-SPONGE” collection).

All isolates from both culture collections were picked and streaked to purity on agar plates. The purified isolates were then grown in Marine Broth (MB) and stocked in fresh MB supplemented with 20% glycerol at −80 °C. For genomic DNA extraction, 2-mL aliquots of shaken cultures (grown in MB medium until stationary phase) of the isolates were centrifuged at 10 000 g for 30 min. Genomic DNA was extracted from the resulting cell pellets using the Wizard® Genomic DNA Purification Kit (Promega, Madison, USA) according to the manufacturer’s instructions. The Purified DNA from all isolates was subjected to 16S rRNA gene amplification by PCR and Sanger sequencing for identification as described in our previous studies [24,25,26,27]. The 16S rRNA gene sequences are deposited at NCBI GenBank, and sequences and accession numbers are available in Tables S1 and S2.

### 4.2. 16S rRNA Gene-Based Phylogenetic Analyses

The 919 isolates from the “MicroEcoEvo” and “EcoTech-SPONGE” culture collections were taxonomically classified based on their 16S rRNA gene sequences using the “RDP Classifier” tool of the Ribosomal Database Project (RDP) v 2.11 [33]. Moreover, using the “Sequence Match” tool of RDP [33], the closest type species of each isolate (see Tables S1 and S2 for details) was recorded. Two separate 16S rRNA gene based phylogenetic trees were generated with MEGAX [89], one for the “MicroEcoEvo” culture collection, consisting of 800 16S rRNA gene sequences and one for the “EcoTech-SPONGE” collection, consisting of 119 16S rRNA gene sequences. Nucleotide sequences of each collection were aligned using the CLUSTALW algorithm. The function “find best DNA/Protein Model (ML)” was employed in MEGAX to determine the evolutionary model that fitted best each dataset. Hence, for the “MicroEcoEvo” collection, a General Time Reversible model with a discrete Gamma distribution (eight categories, +G parameter = 0.5587, [+*I*], 19.25%) was used while for the “EcoTech-SPONGE” collection, a Kimura 2-parameter model, also with a discrete gamma distribution (eight categories, +G parameter = 0.2898, [+*I*], 26.01%), fitted the data best and was employed as evolutionary model. A Maximum-Likelihood (ML) tree was then determined for each collection with bootstrap support using 500 repetitions. Trees with the highest log likelihood (–15928.12 for the “MicroEcoEvo” and –9537.38 for the “EcoTech-SPONGE” collection) were used with a total of 722 (“MicroEcoEvo” collection) and 892 (“EcoTech-SPONGE” collection) nucleotide positions in the final datasets. Both trees were drawn to scale, with branch lengths measured in the number of substitutions per site. In each tree, all positions with less than 85% site coverage were eliminated, i.e., fewer than 15% alignment gaps, missing data, and ambiguous bases were allowed at any position (partial deletion). The calculated trees were visualized and styled in iTOL (Interactive Tree Of Life) v6 [90] and Inkscape [91].

### 4.3. Genome Sequencing and Assembly 

Seventy representative isolates (56 from the “MicroEcoEvo” and 14 from the “EcoTech-SPONGE” collection) had their genomes sequenced and deposited in public databases (See Table S3 for details and accession numbers). Fifty genomes (all from the “MicroEcoEvo” collection) were derived from previous studies and their genome sequencing and assembly procedures have been described elsewhere (e.g., [25,26,63,92,93]). Twenty genomes are original contributions of this study. To sequence these 20 genomes, genomic DNA was extracted from the microbial pellets generated by centrifugation (10 000 g for 30 min) from freshly grown liquid cultures. The Wizard genomic DNA purification kit (Promega, Madison, WI, USA) was used for DNA extraction according to the manufacturer’s instructions. DNA quality and concentration were each assessed with a NanoDrop^TM^ 2000 spectrophotometer (Thermo Fisher Scientific, Waltham, MA, USA) and a Qubit® 4.0 fluorometer (Thermo Fisher Scientific) with the dsDNA BR Assay Kit (Invitrogen, Waltham, MA, USA). DNA integrity was further evaluated by electrophoresis on a 1.2% agarose gel. Genomes were sequenced paired end (2 × 150 bp) on an Illumina NextSeq 550 platform at GENOINSEQ (Biocant, Cantanhede, Portugal). Raw reads were quality filtered, and filtered reads were assembled into contigs using SPAdes v.3.9.0 [94]. Contigs below 1000bp (800bp for *Paraendozoicomonas* sp. Alg238-R29) were removed. 

### 4.4. Comparative Genomics and Phylogenomics Analysis

The 70 genome assemblies were uploaded to the Microbial Genome Atlas (MiGA) and their closest, publicly available, type species were determined using the TypeMat tool of MiGA based on the calculation of average nucleotide identity (ANI) or average amino acid identity (AAI) similarity percentage (see Table S3 for details) [37]. 

Moreover, to ascertain the taxonomic status of difficult-to-classify isolates (e.g., certain *Sphingomonadaceae* and *Rhodobacteraceae* strains of the “MicroEcoEvo” collection), pairwise ANI, and alignment factors (AF; i.e., the fraction of the genome that effectively aligned with the reference genome and served to calculate the ANI) with their respective closest type species were calculated using the Integrated Microbial Genomes & Microbiomes (IMG/M) system from DOE-JGI [95] (see Tables S4 and S5 for details). 

For phylogenomic inference, the genome assembly files of 84 closely related type strains were downloaded from GenBank/NCBI [96]. Phylogenomics analysis of the 154 (70+84) genomes was conducted with the SpeciesTreeBuilder v.01.0 application of the DOE Systems Biology Knowledgebase (KBase) online platform [97] using the function "Insert Set of Genomes into Species Tree", after annotating all genome assemblies with Prokka within KBase. Alignments were based on a set of 49 core genes defined by Clusters of Orthologous Groups of proteins (COGs) families [97]. A maximum likelihood (ML) phylogenomic tree was then constructed using the FastTree2 algorithm [98] and the Jukes-Cantor evolutionary model with 1000 repetitions to estimate bootstrap support values. Graphical visualization and editing of the tree were performed in iTOL v6 [90] and Inkscape [91]. In addition to the phylogenomic tree, a principal coordinates analysis (PCoA) of protein family (Pfam) profiles was performed to examine clustering of the 70 genome-sequenced isolates based on their functional gene content. More information about this analysis is available in the ‘Extended Methodology’ section of Supplementary File S1.

### 4.5. SM-BGC Identification and Network Analysis with antiSMASH and BiG-SCAPE

To identify SM-BGCs, the genome assemblies (fasta-format) of the 70 strains were uploaded to the antiSMASH v. 6.0 [19] online server and analyzed with default settings and all available extra features “on”. Using the “Minimum Information about a Biosynthetic Gene cluster” (MIBiG) repository [19,99], the SM-BGCs identified with antiSMASH were further compared with SM-BGCs encoding the biosynthetic pathways of known compounds in the MIBiG database for the presence of homologous genes (see Tables S7 and S8 for detailed AntiSMASH and MIBiG annotation of SM-BGCs).

To construct sequence similarity networks of the SM-BGCs, the GenBank files from antiSMASH were analyzed with the BiG-SCAPE software [38] using default parameters. BiG-SCAPE defines a distance metric between gene clusters based on a comparison of their protein domain content, order, copy number and sequence identity [37], grouping SM-BGCs that share ≥70% similarity into Gene Cluster Families (GCF) [38]. The resulting SM-BGC networks were edited in Inkscape [91]. 

### 4.6. Marine Bacterial Isolates and Test Strains Used in Antimicrobial Assays

The 70 genome-sequenced isolates were revived from glycerol stocks by inoculating 50 µl of the stock solution into 10 ml of half strength MB (1:2 diluted) medium (20.05 g L^−1^ MB (Carl Roth GmbH + Co. KG, Karlsruhe, Germany) dissolved in a 1:1 *v/v* ratio of dH_2_O: artificial seawater (ASW)). The salt composition of ASW was as follows: 23.38 g L^−1^ NaCl, 2.41 g L^−1^ MgSO_4_.7H_2_O, 1.90 g L^−1^ MgCl_2_.6H_2_O, 1.11 g L^−1^ CaCl_2_.2H_2_O, 0.75 g L^−1^ KCl, 0.17 g L^−1^ NaHCO_3_. The purity of all cultures was confirmed by streaking them on half strength MA plates. 

For the antifungal assays, the wild-type *Candida glabrata* KCHR606 [39], and *Δpdr1* complete deletion mutant strains [40], kindly provided by Hiroji Chibana (Chiba University, Japan), *C. albicans* ATCC SC5314, *C. auris* ATCC B11220 and *C. parapsilosis* ATCC 22019 were used. For the antibacterial assays, *Staphylococcus aureus* ATCC 6538, methicillin-resistant *Staphylococcus aureus* JE2 (NR-46543, B.E.I Resources – Part of ATCC), *Salmonella enterica* subsp. *enterica* serovar Typhimurium DSM 24522 and *Vibrio parahaemolyticus* DSM 101031 were used. The test strains were grown and maintained in appropriate liquid media as follows: Yeast extract Peptone Dextrose (YPD) agar [20 g L^−1^ D(+)-glucose (anhydrous), 20 g L^−1^ yeast extract, 20 g L^−1^ agar, 10 g L^−1^ peptone] for *Candida* strains; Tryptic Soy Broth (TSB) for the human-pathogenic bacteria; full-strength MB for *V. parahaemolyticus*. The incubation temperatures were 37 °C for all human-pathogenic bacteria, 30 °C for the *Candida* strains and 24 °C for *V. parahaemolyticus* DSM 101031. 

### 4.7. Cross-Streak Assays

The cross-streak assay is an antimicrobial activity screening technique that delivers qualitative or semi-quantitative results on the inhibitory activities of a certain prokaryotic isolate against a given test strain (usually also a prokaryote or a yeast) [41]. Thirty microliters of each marine bacterial isolate grown in MB were spread on 1.5% agar plates as a two-cm-wide, vertical line dividing the plate into two equal-sized halves (see scheme in Figure S2a,b). The medium content of these agar plates varied according to the test microorganisms used: full-strength MA supplemented with 0.5% (*v/v*) of glucose was used for *Candida* strains; Mueller–Hinton agar (MHA) prepared with 2% ASW was used for the pathogenic bacteria. If the marine bacterial isolates did not grow well on MHA, full strength MA was used instead. After an incubation period of 5 days at 24 °C, 5 µl of overnight-grown liquid cultures of the pathogen were placed close to the central line, ensuring the absence of contact between the different strains. For homogeneous seeding, the test strain was streaked perpendicular to the central line with an inoculation loop (Figure S2a), first toward the border of the plate and subsequently inwards. The overnight-grown liquid cultures of the pathogens were prepared as follows: pre-inoculates of the five *Candida* strains were prepared in YPD liquid medium and grown overnight. The next day, the optical densities (OD) of the *Candida* pre-cultures were determined at 600 nm, the cultures were diluted in YDP to an OD of 0.1 and incubated for five to six hours until mid-exponential phase. Before inoculation onto the cross-streak assay plates, the ODs of the *Candida* cultures were again adjusted to 0.1, and 5 µl of each cell suspension was then streaked onto the cross-streak plates (Figure S2a). For the antibacterial assay, overnight liquid cultures of *S. aureus*, MRSA, and *S. enterica* serovar Typhimurium were prepared in TSB and of *V. parahaemolyticus* in MB. Thereafter, 5 µl of these liquid cultures were spread onto the cross-streak plates (Figure S2b) as described above. After further incubation of the cross-streak assay plates (48 h for fungal pathogens and 24 h for bacterial pathogens) at 30 °C, the overall growth of test strains and the size of inhibition zones was evaluated. All experiments were performed at least in triplicate for each marine bacterium—pathogen pair. Agar plates inoculated only with the pathogens were used as negative controls (Figure S2a,b). Inhibitions were ranked as: (1.0 point) complete inhibition, no pathogen growth compared with controls, Figure S2c); (0.75 of a point) strong inhibition, ca. 75% growth reduction, Figure S2d; (0.5 of a point) moderate inhibition, ca. 50% growth reduction, Figure S2e); (0.25 of a point) weak inhibition, ca. 25% growth reduction compared with controls, Figure S2f); (0 points) no inhibition, normal growth of pathogens, similar to controls. 

## 5. Conclusions

The present study explored two comprehensive culture collections of marine host-associated bacteria for their secondary metabolite biosynthesis capacities and antimicrobial activities against human pathogenic bacteria and *Candida* species known to be increasingly resistant to currently marketed drugs. It revealed the secondary metabolite coding potential and bioactivities of 70 marine host-associated bacteria, several of which belonged to understudied or novel species and genera. The here analyzed strains possessed rich and varied secondary metabolite biosynthesis potential, with many SM-BGCs having low similarity to SM-BGCs encoding known compounds, thus suggesting biosynthetic novelty. Multiple isolates showed antimicrobial activities, with *Exiguobacterium* sp. CinTr1, *Aquimarina* sp. EL33, Aq135 and Aq349, unclassified *Flavobacteriaceae* sp. RHTr2, *Halomonas* sp. Alg239-R46, unclassified *Rhodobacteraceae* sp. Alg231_04, *Sulfitobacter* sp. EL44 and *Shimia* sp. Alg240-R146 standing out as the most promising producers of antifungal and/or antibacterial compounds. In conclusion, the marine bacterial culture collections described here constitute a valuable resource for bioprospecting natural products and/or enzymes of pharmaceutical or biotechnological interest. Interestingly, despite extensive efforts during the last decades to isolate and screen microbial strains from marine invertebrates for pharmaceutical-biotechnological applications, our results showed that sponges and octocorals still represent an untapped resource of hitherto unknown bacterial taxa carrying genes coding for novel natural products. Beyond their potential for the blue economy sector, the microorganisms described here are also a great resource and toolbox to study the ecology and mechanisms of host-microbe interactions, and their suitability for employment as beneficial microbes for marine animals to maintain, improve and/or restore host and reef ecosystem health. 

## Data Availability

All 16S rRNA gene and whole genome sequences of this study are deposited at NCBI GenBank and European Nucleotide Archive (ENA), respectively. The 20 new genome sequences original to this study were deposited at ENA under project accession numbers PRJEB50695 and PRJEB50698. 16S rRNA gene accession numbers can be found in Table S1 (“MicroEcoEvo” collection) and Table S2 (“EcoTech-SPONGE” collection). Genome assembly accession numbers can be found in Table S3.

## Supplementary Materials

The following supporting information can be downloaded at: https://www.mdpi.com/article/10.3390/md21010034/s1. Supplementary file S1: This file contains an extended methodology and result section on the phylogenomics assessment and the principal coordinates analysis (PCoA) of the functional (Pfam) profiles of the 70 genomes analyzed in this study, as well as supplementary figures S1 and S2: S1: Multivariate analysis of the Pfam profiles of the 70 genomes. S2: Schematic representation and examples of the antimicrobial cross-streak assays. Supplementary file S2: This file contains all Supplementary tables (S1−S10): S1 and S2: Complete list of isolates belonging to the “MicroEcoEvo” and “EcoTech-SPONGE” culture collections, respectively. S3: General genomic features, genome assembly accession numbers and quality metrices of the 70 genome sequenced isolates. S4: ANI and AAI similarity of selected, genome-sequenced isolates with the genomes of closest type species, according to MiGA. S5: ANI and AF vales of genomes of difficult-to-classify alphaproteobacterial isolates to genomes of related type species, according to DOE-JGI IMG/M system. S6: Protein family (Pfam) based functional annotation of the 70 genome-sequenced isolates. S7: antiSMASH results – SM-BGC annotation for the 70 genome sequenced isolates. S8: SM-BGCs with >70% similarity to SM-BGCs of known compounds present in the MIBiG database. S9: Overview on the gene cluster families (GCF) shown in Figure 5B and details on the origin (isolate) and compound class contributing to each GCF. S10: Pairwise comparison of the 16S rRNA gene sequences of the 14 *Pseudoalteromonas* isolates from the “EcoTech-SPONGE” culture collection with those of the SM-BGC enriched *Pseudoalteromonas* clade reported in Chau et al., 2021 [62].
