# Supplementary material for: Marine Sponge and Octocoral-Associated Bacteria Show Versatile Secondary Metabolite Biosynthesis Potential and Antimicrobial Activities against Human Pathogens
Source: Mar Drugs. 2022 Dec 30;21(1):34. doi: 10.3390/md21010034 (PMC9860996; doi:10.3390/md21010034)
Supplement: Supplementary file 1 [file marinedrugs-21-00034-s001.zip › Supplementary_file_S1_revised.pdf]

Supplementary File S1

Marine Sponge and Octocoral-Associated Bacteria Show Versatile Secondary Metabolite Biosynthesis Potential and Antimicrobial Activities Against Human Pathogens

João F. Almeida [1,2], Matilde Marques [1,2], Vanessa Oliveira [3], Conceição Egas [4,5], Dalila Mil-Homens [1,2], Romeu Viana [1,2], Daniel F. R. Cleary [3], Yusheng M. Huang [6], Arsénio M. Fialho [1,2], Miguel C. Teixeira [1,2], Newton C. M. Gomes [3], Rodrigo Costa [1,2,7,*] and Tina Keller-Costa [1,2,*]

1 iBB—Institute for Bioengineering and Biosciences and i4HB—Institute for Health and Bioeconomy, Instituto Superior Técnico, Av. Rovisco Pais, 1049-001 Lisbon, Portugal
2 Department of Bioengineering, Instituto Superior Técnico, Av. Rovisco Pais, 1049-001 Lisbon, Portugal
3 Department of Biology and Centre for Environmental and Marine Studies (CESAM), University of Aveiro, 3810-193 Aveiro, Portugal
4 Center for Neuroscience and Cell Biology (CNC), Rua Larga—Faculdade de Medicina, University of Coimbra, 3004-504 Coimbra, Portugal
5 Biocant—Transfer Technology Association, BiocantPark, 3060−197 Cantanhede, Portugal
6 Department of Marine Recreation, National Penghu University of Science and Technology, Magong City 880-011, Taiwan
7 Centre of Marine Sciences (CCMAR/CIMAR LA), University of Algarve, Campus de Gambelas, 8005−139 Faro, Portugal
***** Correspondence: rodrigoscosta@tecnico.ulisboa.pt (R.C.); tinakellercosta@tecnico.ulisboa.pt (T.K.-C.); Tel.: (+351)-21-841-7339 (R.C.); (+351)-21-841-3167(T.K.-C.)

Keywords: bioprospection; biosynthetic gene clusters; blue biotechnology; culture collections; genomics; marine bacteria

Extended Methodology

Principal coordinate analysis (PCoA) of functional profiles

Protein family (Pfam) profiles were obtained for the 70 genome assemblies using our in-house, automated genome annotation pipeline MeLanGE, fully documented and available on GitHub: https://sandragodinhosilva.github.io/MeLanGE. Briefly, all genomes (contig fasta files) were first annotated with Prokka v1.14.6 [1] to obtain GenBank format and amino acid fasta files. Thereafter, proteins were queried against the Pfam database [2]. The resulting Pfam count table was Hellinger-transformed (i.e., by calculating the square-root of the relative abundance of Pfam entries) to normalize the dataset. The Bray-Curtis dissimilarity index was then employed to compute a distance matrix of the Pfam profiles of all genomes and to perform a Principal Coordinates Analyses (PCoA) in PAST v4.05 [3]. The resulting PCoA graph was edited in Inkscape [4]. See also **Table S6** for the Pfam counts for each genome.

Extended Results

Phylogenomics analysis of *Alphaproteobacteria* isolates from the MicroEcoEvo" collection

The three *Sphingorhabdus* genomes of the "MicroEcoEvo" collection, i.e., strains EL138, Alg231_15 and Alg239-R122, formed a clade with *Parasphingorhabdus marina* (GCA_900128895) and *P. litoris* (IMG genome ID 2574179751) instead of clustering with members of the *Sphingorhabdus* genus (**Figure 3**). Both Alg231_15 and EL138 shared about 75% AAI with *P. marina* and *P. litoris* and only around 60% AAI with *Sphingorhabdus contaminans* (GCA_007280415.1) and *S. wooponensis* (GCA_003933235.1) (**Table S4**). Moreover, both strains shared > 81% ANI and an alignment factor (AF) around 70%, with *P. litoris*, whereas with *S. contaminans* and *S. wooponensis*, ANI values were only around 73%, and AFs were only 11 and 8%, respectively (**Table S5**). These data support the reclassification of strains EL138 and Alg231_15 into *Parasphingorhabdus*. The taxonomic placement of strain Alg239-R122 remained unresolved as it did not share high enough AAI, ANI and AF values that would confirm classification into either *Sphingorhabdus*, *Parasphingorhabdus* or *Blastomonas* (**Tables S4** and **S5**).

The unclassified *Rhodobacteraceae* sp. strain EL129 ("MicroEcoEvo" collection) clustered together with *Roseovarius marisflavi* (GCA_900142625.1) (AAI 70.37%) and *Pelagicola litorisediminis* (GCA_900172295) (AAI 72.33%) (**Figure 3**) and shared about 70% AAI with other *Roseovarius* type genomes, suggesting the strain could belong either to the genus *Roseovarius* or *Pelagicola* (**Table S4**). Unclassified *Rhodobacteraceae* strains Alg231_04 and EL27 from the "MicroEcoEvo" collection clustered within the *Leisingera-Phaeobacter-Pseudophaeobacter* clade (**Figure 3**). Indeed, strain EL27 shared highest ANI (85.29%), AAI (87.38%) and AF (70.455%) values with *Pseudophaeobacter arcticus* (GCA_000473205) (**Table S4** and **S5**), placing strain EL27 into the *Pseudophaeobacter* genus. Although strain Alg231_04

shared around 73% AAI with several *Leisingera* species, AFs were all below 50%, leaving the taxonomic status of this strain inconclusive (**Tables S4** and **S5**). Finally, unclassified *Rhodobacteraceae* sp. strains EL53, isolated from the octocoral *Eunicella labiata,* and Alg241-R94, isolated from the marine sponge *Spongia officinalis,* formed a well-supported, tight clade within the genus *Ruegeria* (**Figure 3**) and shared 97.7% ANI and 90% AF with each other, indicating that both strains belong to the same species **(Table S5**). Both strains shared > 76% AAI with *Ruegeria atlantica* (GCA_001458195), which suggests that they could belong to the genus *Ruegeria*, although the AFs were below 40% (**Tables S4** and **S5**).

Multivariate analysis of functional gene profiles of genome-sequenced isolates

To assess the functional and phylogenetic relatedness among the 70 genome-sequenced strains, a multivariate, principal coordinates analysis (PCoA) using protein family (Pfam) annotations of the 70 genomes was performed. The PCoA easily distinguished between bacterial classes (**Figure S1**), showing that the functional gene profiles follow overall strain phylogeny. The *Gammaproteobacteria* genomes of this study were functionally diverse, scattering along the bottom left quarter of the ordination space. Notably, unclassified *Oceanospirillales* sp. strain XeTr1 was close to *Paraendozoicomonas* sp. strain Alg238-R29 in the ordination space, congruent with our phylogenomics analysis (**Figure 3**). The *Alphaproteobacteria* genomes formed two clusters, separated along coordinate 2, one comprising all *Rhodobacteraceae* genomes, and one with the *Sphingomonadales* genomes (**Figure S1**). Within the *Rhodobacteraceae* cluster, *Ruegeria* sp. strains Alg240_R139 and Alg239_R130 were in proximity to unclassified *Rhodobacteraceae* sp. strains EL53 and Alg241_R94, corroborating the phylogenomics inference (**Figure 3**) and AAI values, which inserted both genomes in the *Ruegeria* genus. The *Flavobacteria* and *Cytophagia* (both *Bacteroidetes*) strains were found in proximity to each other in the ordination space and well separated from other classes along coordinate 2. Interestingly, unclassified *Flavobacteriaceae* sp. strain RHTr2 and *Aquimarina* sp. strain Aq107 clustered tightly together, suggesting that the functional gene content of strain RHTr2 is quite similar to that of the *Aquimarina* genus (**Figure S1**).

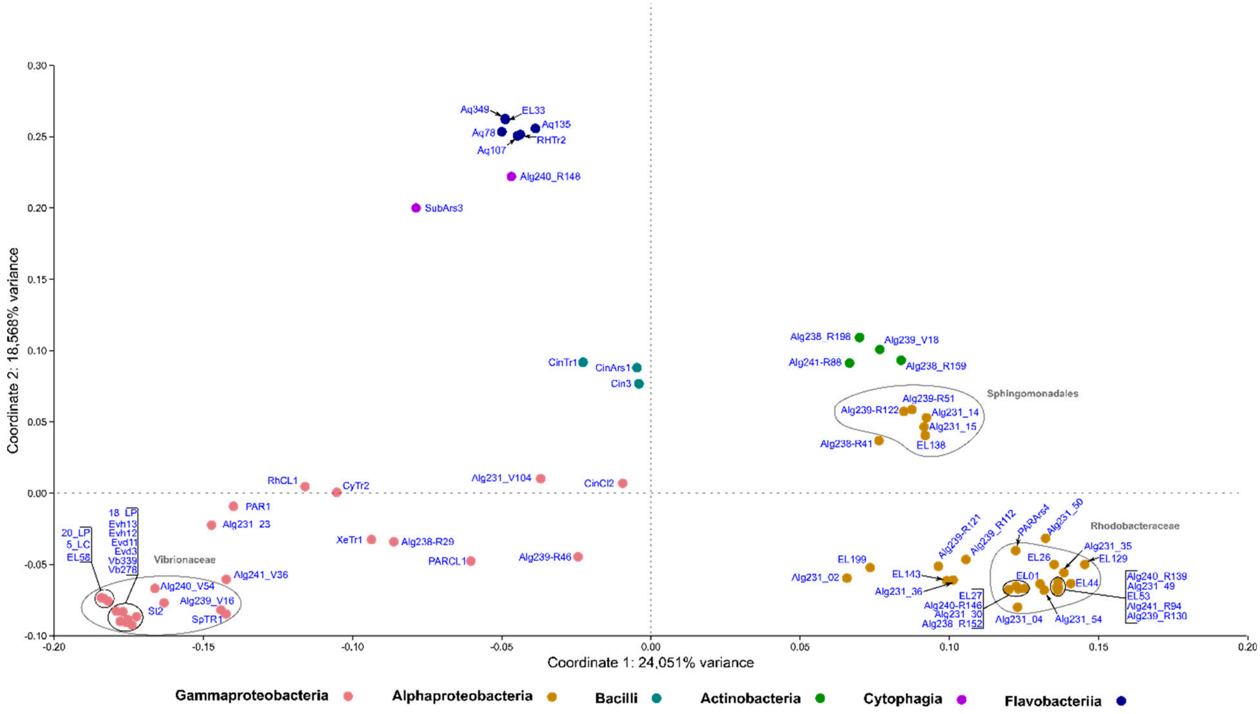

Supplementary figure S1: Multivariate analysis of the protein family (Pfam) profiles of the 70 genome-sequenced isolates. The principal coordinates analysis (PcoA) was calculated from Hellinger-transformed Pfam abundance data. The ordination is drawn in Eigenvalue scale. Class-level taxonomy of each bacterium is indicated by the colouring of each circle and each circle represents one genome. Salmon – *Gammaproteobacteria*; Dark yellow – *Alphaproteobacteria*; Teal – *Bacilli*; Green – *Actinobacteria*; Purple – *Cytophagia*; Blue – *Flavobacteriia.*

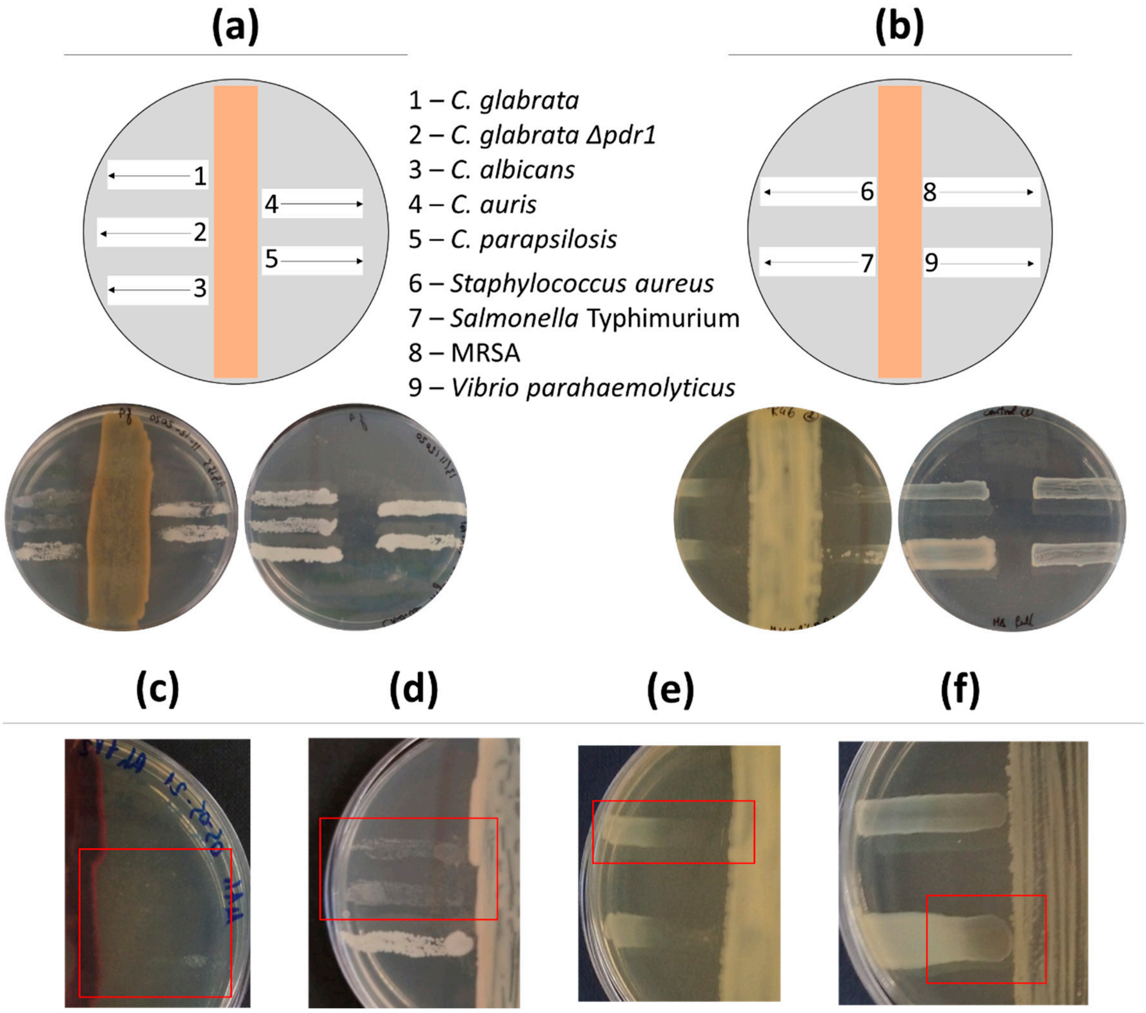

Supplementary figure S2: Schematic representation, example, and control (pathogens only) plate of an **(a)** antifungal and (**b)** antibacterial cross-streak assay. **(c)** Example of a complete inhibition of *Candida auris* and *C. parapsilosis* by *Flavobacteriaceae* sp. strain RHTr2 (1.0 point). **(d)** Example of a strong inhibition of *Candida glabrata* by *Shimia* sp. strain Alg231-30 (0.75 of a point). **(e)** Example of a moderate inhibition of *Staphylococcus aureus* by *Halomonas* sp. Alg239-R46 (0.5 of a point). **(f)** Example of a weak inhibition of *Salmonella* Typhimurium by *Oceanospirillales* sp. XeTr1 (0.25 of a point).

References

1. Seemann, T. Prokka: Rapid Prokaryotic Genome Annotation. *Bioinformatics* **2014**, *30*, 2068–2069, doi:10.1093/bioinformatics/btu153.
2. El-Gebali, S.; Mistry, J.; Bateman, A.; Eddy, S.R.; Luciani, A.; Potter, S.C.; Qureshi, M.; Richardson, L.J.; Salazar, G.A.; Smart, A.; et al. The Pfam Protein Families Database in 2019. *Nucleic Acids Research* **2019**, *47*, D427–D432, doi:10.1093/nar/gky995.
3. Hammer, Ø.; Harper, D.; D. Ryan, P. PAST: Paleontological Statistics Software Package for Education and Data Analysis. *Palaeontologia Electronica* **2001**, *4*, 9.
4. Inkscape Project Inkscape Available online: https://inkscape.org (accessed on 26 August 2021).
